# Quantitative proteomic analysis and verification identify global protein profiling dynamics in pig during the estrous cycle

**DOI:** 10.3389/fvets.2023.1247561

**Published:** 2023-09-28

**Authors:** Haiyun Xin, Baohong Li, Fanming Meng, Bin Hu, Sutian Wang, Ying Wang, Jianhao Li

**Affiliations:** ^1^State Key Laboratory of Swine and Poultry Breeding Industry, Guangdong Key Laboratory of Animal Breeding and Nutrition, Institute of Animal Science, Guangdong Academy of Agricultural Sciences, Guangzhou, China; ^2^Maoming Branch, Guangdong Laboratory for Lingnan Modern Agriculture, Maoming, China; ^3^Guangzhou Customs Technical Center, Guangzhou, China

**Keywords:** sow, estrus detection, saliva, quantitative proteomics, differentially expressed proteins, reproduction efficiency

## Abstract

The current estrus detection method is generally time-consuming and has low accuracy. As such, a deeper understanding of the physiological processes during the estrous cycle accelerates the development of estrus detection efficiency and accuracy. In this study, the label-free acquisition mass spectrometry was used to explore salivary proteome profiles during the estrous cycle (day −3, day 0, day 3, and day 8) in pigs, and the parallel reaction monitoring (PRM) was applied to verify the relative profiles of protein expression. A total of 1,155 proteins were identified in the label-free analysis, of which 115 were identified as differentially expressed proteins (DEPs) among different groups (*p* ≤ 0.05). Functional annotation revealed that the DEPs were clustered in calcium ion binding, actin cytoskeleton, and lyase activity. PRM verified the relative profiles of protein expression, in which PHB domain-containing protein, growth factor receptor-bound protein 2, elongation factor Tu, carboxypeptidase D, carbonic anhydrase, and trefoil factor 3 were confirmed to be consistent in both label-free and PRM approaches. Comparative proteomic assays on saliva would increase our knowledge of the estrous cycle in sows and provide potential methods for estrus detection.

## Introduction

As an indispensable part of sow reproductive management, prompt and accurate estrus detection directly determines the overall productivity of sow utilization by affecting the optimal insemination moment, reducing ineffective feeding, and timely eliminating sows with low reproductive performance (1). Several methods have been applied to detect estrus in sows, for instance, behavioral observation, teaser boar test, and steroids estimation (2). The most common method is manual recognition based on swelling of the vulva and behavioral signs to a back-pressure test or boar. The observation method is time-and labor-intensive, while accuracy depends heavily on the experience of the technician (2). Few steroids are present in boar saliva and are considered to contribute greatly to promoting estrus behaviors in sows (3). In this context, a boar saliva analog mixture of androstenone, androstanol, and quinoline was synthesized and shown effective for estrus detection (4, 5). As a steroid pheromone, the analog protects sows from boar exposure and probable disease infection by teaser boar (6, 7).

With the development of artificial intelligence, many researchers have focused on the automatic identification of sow estrus by sensing regular temperature changes (8, 9), activity (10) or the frequency and duration of the sow’s visit to a boar (11) after gathering massive images or videos (12, 13). These automatic identifications showed relatively lower or equivalent accuracy compared with manual observation (9, 11). A study conducted by Lei (11) first combined machine vision with a bionic boar model to identify sows in heat. Despite the high cost of machines, recognition has a high accuracy of 98.25% and other advantages, such as greater intelligence and biosafety, but less stress than manual detection methods. Estimations based on physical or biochemical parameters of vaginal mucus (14), hormone concentration or crystal forms in body fluids (15, 16) have also been investigated, but they were not widely used because of the high cost of assay kits or their unstable characteristics. It remains a vital concern for the breeding industry to explore timely, efficient, and sensitive estrus diagnostic methods. Meanwhile, knowledge gaps regarding estrous cycles physiology must be addressed.

As an important body secretion, saliva is rich in steroid hormones, nucleic acids, proteins, and polypeptides, portions of which can be passively diffused or actively transported from the blood, revealing high similarity with blood (17–19). Recently, saliva has become an effective, noninvasive, easily available, and convenient material for detecting the physiological status of animals. Saliva estimation has great application prospects for estrus detection (2). Herein, we compared salivary proteomics in sows during the estrous cycle and verified the relative expression of several proteins to supplement our understanding of estrus physiology and provide a reference for estrus recognition development.

## Materials and methods

### Animals and saliva collection

Eight healthy primiparous hybrid sows (Duroc × Tibetan crossbred pig) showing normal estrus were selected for this study. Animal welfare in this study was safeguarded and approved by the Animal Care and Use Committee of Guangdong Academy of Agricultural Sciences (Protocol number:2018001). The experimental sows were observed daily in the morning and evening to assess their estrus status. Estrus identification was conducted by external observation and the back pressure reaction. A sow standing still and accepting a boar crawling with a swelling vulva was considered estrus, and the day of estrus was defined as day 0. Saliva, representing four stages of the estrous cycle, was collected every morning before feeding, including ED-3 (proestrus, *n* = 3): 3 days before estrus; ED0 (estrus, *n* = 3): the day of estrus; ED3 (metestrus, *n* = 3): 3 days after estrus; and ED8 (diestrus, *n* = 3): 8 days after estrus. The number of saliva collections for each timepoint were three. Pigs were curious and chewed the hanging cotton bags wrapping up with absorbent cotton balls. Saliva infiltrated the absorbent cotton ball was squeezed out, released into the sample bag, and then transferred into clean tubes. The samples were subsequently centrifugated at 4000 × *g* for 5 min at 4°C and the supernatant was mixed with Protease Inhibitor Cocktail (10 μL/mL, Sigma) before storage at −80°C until quantitative proteomics assay.

### Protein extraction, quality test, digestion and desalination

Protein extraction and LS-MS analysis were conducted by Novogene Co., Ltd. The sample was lysed with DB lysis buffer (8 M Urea, 100 mM TEAB, pH 8.5, Sigma) in a 1.5 mL centrifuge tube, and the lysate was centrifuged at 12000 × *g* for 15 min at 4°C. The supernatant was reduced with 10 mM DTT (Sigma) at 56°C for 1 h and alkylated with sufficient IAM (Sigma) for 1 h at room temperature in the dark. The total protein concentration was determined using the Bradford protein quantitative kit (Beyotime). Then, 20 μg of protein was loaded into each well for gel electrophoresis to assess protein quality. Next, 120 μg of protein samples were mixed with DB lysis buffer (8 M Urea, 100 mM TEAB, pH 8.5, Sigma), trypsin (Promega) and 100 mM TEAB buffer to make the final volume up to 100 μL. The mixture was incubated for 4 h at 37°C. Subsequently, trypsin and CaCl_2_ (Sinopharm chemical reagent Co., Ltd) were added, and the samples were incubated overnight. Formic acid (Thermo Fisher Scientific) was added to the digested samples, adjusted to pH < 3, and centrifuged at 12000 × *g* for 5 min at room temperature. The supernatant was loaded onto a C18 desalting column (Thermo Fisher Scientific), washed three times with washing buffer (0.1% formic acid, 3% acetonitrile, Thermo Fisher), eluted with elution buffer (0.1% formic acid, 70% acetonitrile), and the eluents of each sample were collected and lyophilized.

### LC–MS analysis

The lyophilized proteins were dissolved in 10 μL of mobile phase A (0.1% formic acid in distilled deionized water), centrifuged at 14,000 × *g* for 20 min at 4°C, and 1 μL of supernatant was loaded into a homemade C18 Nano-Trap column (4.5 cm × 75 μm, 3 μm). Proteins were then linearly gradient fractionated using a homemade analytical column (15 cm × 150 μm, 1.9 μm) with mobile phases A and B (80% acetonitrile, 0.1% formic acid) and analyzed using a Q Exactive™ HF-X mass spectrometer (Thermo Fisher Scientific). Ions source of Nanospray Flex™ (ESI) were fully scanned, ranging from 350 to 1,500 m/z, in which the top 40 precursors of the highest abundance were fragmented by higher energy collisional dissociation (HCD) and analyzed by MS/MS using the following parameters: the resolution was 15,000 (at m/z 200), the automatic gain control (20) target value was 1 × 10^5^, the maximum ion injection time was 45 ms, the normalized collision energy was set at 27%, the intensity threshold was 2.2 × 10^4^, and the dynamic exclusion parameter was 20 s.

### Identification and quantitation of proteins

The offline spectra were searched using Proteome Discoverer 2.2 (PD 2.2, Thermo Fisher Scientific), with a maximum of two missed cleavage sites allowed. Peptides with credibility greater than 99% were considered peptide spectrum matches (PSMs). A protein containing at least one unique peptide was defined as trusted. These preserved PSMs and trusted proteins were subjected to false discovery rate (FDR) analysis to remove data larger than 0.01. The general distribution character of all the identified proteins were analyzed with Jvenn online program.^1^ Relative quantitative differences were analyzed using the t-test. Proteins with a fold change ≥1.5 or ≤ 0.67 and *p* ≤ 0.05 were defined as DEPs.

### Bioinformatics analysis

Both identified proteins and DEPs were subjected to bioinformatics analysis. Functional prediction of Gene Ontology (GO) and InterPro (21) was conducted using an interproscan program (version 5.22–61.0) against the non-redundant protein databases, including Pfam, PRINTS, ProDom, SMART, ProSite, and PANTHER (22). Protein family and pathway predictions were analyzed using the Clusters of Orthologous Groups (23) and Kyoto Encyclopedia of Genes and Genomes (KEGG) databases. Potential protein–protein interactions were assayed using the STRING-db server (24).^2^

### Validation of relative expression level by PRM

To verify the relative expression levels of DEPs identified in label-free quantitative proteomic analysis, saliva samples collected on days −3, 0,3, 8 for each day (*n* = 3) during the estrous cycle were confirmed by PRM with an acquired MS/MS spectrum. Proteins extracted from saliva were quantity and quality tested, enzymatically hydrolyzed, desalted, and lyophilized, as described above. For LC–MS/MS analysis pre-experiment, 1 μg of the mixture was eluted as a “label-free” method using the EASY-nLCTM1200 UHPLC system (Thermo Fisher Scientific) coupled with a Q Exactive series mass spectrometer (Thermo Fisher Scientific). The raw data were searched by PD2.2 software with full scan mode, and PRM pattern sequentially, and the selected peptides were determined using Skyline software based on reproducibility and stability. In the LC–MS/MS formal experiments, equivalent peptides pretreated with trypsin were spiked with the same amount of isotope-labeled peptide as an internal standard. Samples were analyzed by full scan and PRM patterns as described above. For offline data analysis, the peak area of each target protein was corrected according to the internal standard peptide to make it available for subsequent evaluation of relative abundance.

### Data analysis

The relative expression levels of proteins were represented by fold changes by comparing the abundance of proteins during the estrous cycle. Statistical analysis was carried out by GraphPad Prism, version 8. Data comparisons between two groups were analyzed using the t-test. Data comparisons among three or more groups were carried out using one-way ANOVA, followed by Tukey multiple range test. Statistical significance was set at *p* < 0.05.

## Results

### Identification of salivary proteins during the estrous cycle

**Figure 1:**
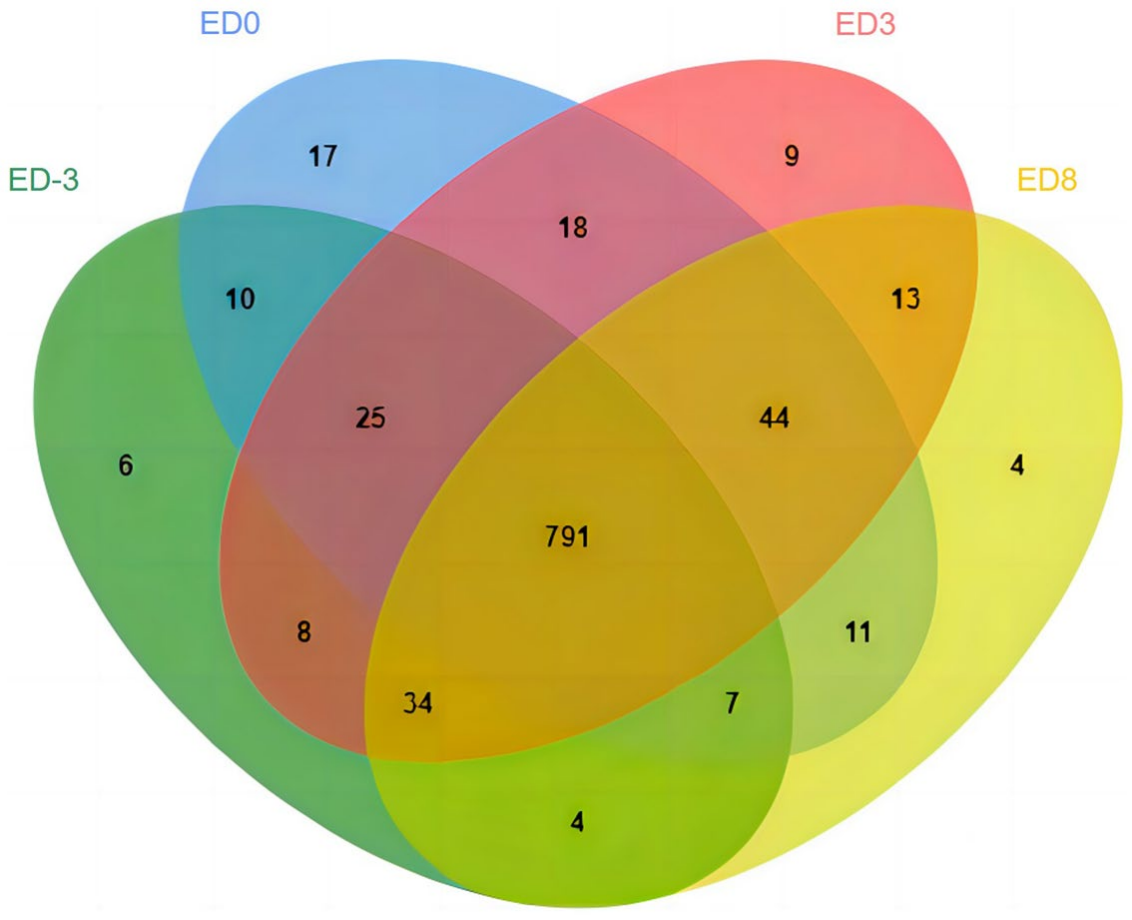
Venn diagram of identified proteins at proestrus (ED-3), estrus (ED0), metestrus (ED3), and diestrus (ED8) stages in pig saliva.

A total of 8,091 peptides and 1,155 proteins were identified in salivary samples, including 885, 923, 942, and 908 proteins in the ED-3, ED0, ED3, and ED8 groups, respectively. The general distribution character of all the identified proteins were analyzed with Jvenn online program, as shown in Figure 1. The number of shared proteins in four groups was 791, and the number of specific proteins for ED-3, ED0, ED3, and ED8 were 6, 17, 9, and 4, respectively (Figure 1).

### Functional prediction of total proteins

**Figure 2:**
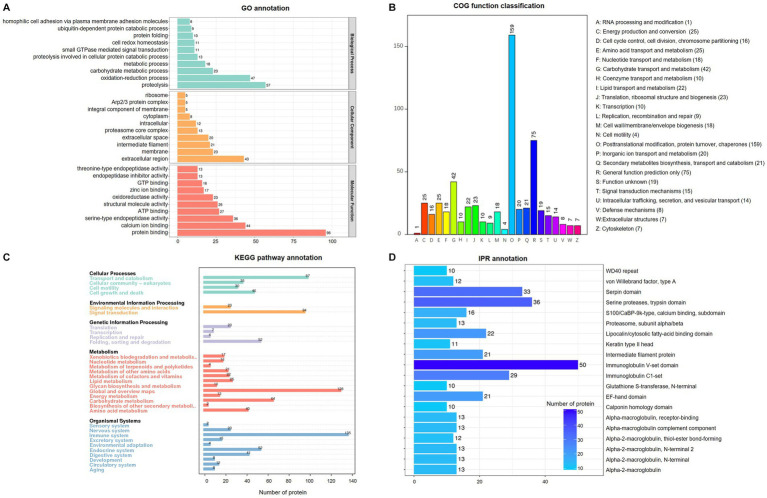
Enrichment analysis of identified total proteins in pig saliva during the estrous cycle. **(A)** Gene ontology (GO) analysis; **(B)** Clusters of Orthologous Groups of proteins enrichment; **(C)** Kyoto Encyclopedia of Genes and genomes (KEGG) pathway annotation. **(D)** InterPro (IPR) functional analysis.

Functional prediction and classification of total proteins were conducted using the interproscan program, as shown in Figure 2. The results of GO enrichment indicated that all proteins were classified into three domains:666 proteins in molecular function, 399 proteins in biological processes, and 202 proteins in cellular component (Figure 2A). Molecular function had the largest number of items, and the top three items were protein binding (*n* = 96), calcium ion binding (*n* = 44), and serine-type endopeptidase activity (*n* = 36). The biological process results indicated that proteins were involved in proteolysis (*n* = 57), oxidation–reduction process (*n* = 47) and carbohydrate metabolic process (*n* = 23). According to cellular component results, most proteins are involved in the extracellular region (*n* = 43), membrane (*n* = 23), intermediate filament (*n* = 21) and extracellular space (*n* = 20). For COG analysis (Figure 2B), the top three functional classes were classified as O (posttranslational modification, protein turnover, chaperones), R (General function prediction only), and G (carbohydrate transport and metabolism) with 159, 75, and 42, respectively. KEGG analysis (Figure 2C) revealed that most proteins were involved in metabolism (*n* = 362), organismal (*n* = 300), and cellular processes (*n* = 208), while environmental information processing (*n* = 117) and signal transduction (*n* = 94) enriched fewer proteins. In the IPR prediction (Figure 2D), the immunoglobulin V-set domain (*n* = 50) contained the most proteins.

### Functional annotation and prediction of DEPs

**Figure 3:**
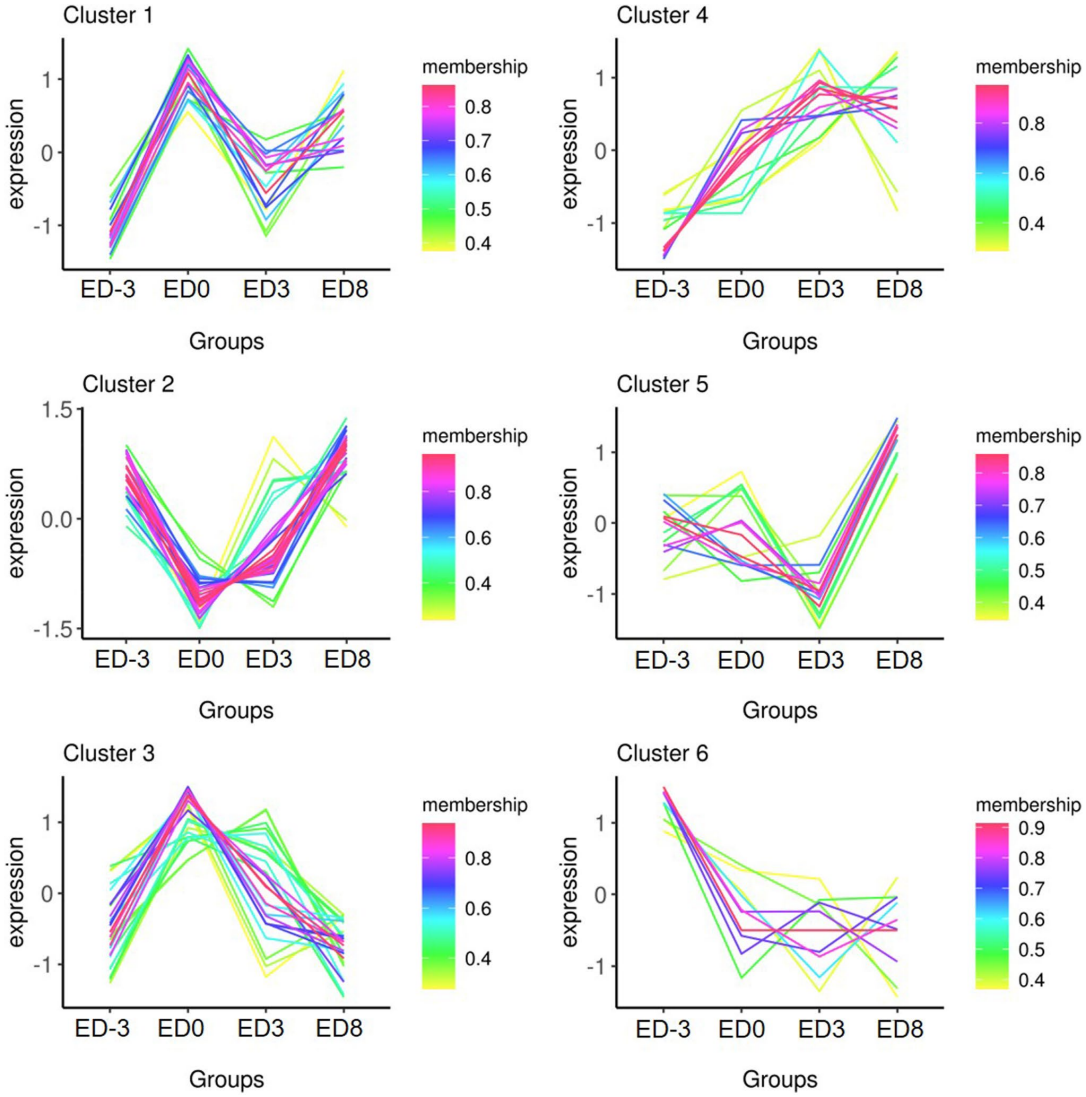
Time- series expression trends of six clusters of DEPs in pig saliva during the estrous cycle.

Protein quantity was compared among the different groups to isolate DEPs (FC ≥ 1.5, *p* ≤ 0.05). In total, 93 proteins were identified as DEPs, of which the expression of 38 proteins were upregulated and 83 were downregulated. The relative fold changes are shown in Tables 1, 2. The comparison ED3 vs. ED8 and ED0 vs. ED8 showed the largest number of proteins with downregulated expressions, with 30 and 34, respectively. Most proteins with upregulated expressions came from groups ED0 vs. ED-3 and ED3 vs. ED8 (15 and 11, respectively). In ED0 vs. ED-3 and ED0 vs. ED8, the expressions of 20 proteins were upregulated, and those of 12 were downregulated with FC > 2. All DEPs were divided into six sets according to time-series expression patterns, in which 21, 14, 10, 36, 17, and 17 proteins were clustered (Figure 3).

**Table 1:** List of upregulated DEPs in different comparisons.

No.	Protein	Description	Gene	FC					
				ED0/ED-3	ED0/ED3	ED0/ED8	ED-3/ED3	ED-3/ED8	ED3/ED8
1	A0A287AZK2	Transgelin	TAGLN				Inf		
2	A0A287AW99	Intelectin 2	ITLN2	Inf					
3	A0A480NC04	Proteasome subunit beta		Inf					
4	F1RKM0	Lamin B1	LMNB1	Inf					
5	A0A076KWW8	C-type lectin domain family 8 member A	CLEC8A	4.37					
6	A0A4X1UP22	PHB domain-containing protein		2.82					
7	A0A4X1W7U1	Peptidase S1 domain-containing protein		2.53					
8	A0A4X1UBJ8	Uncharacterized protein		2.23					
9	B6E241	Growth factor receptor bound protein 2	GRB2	2.14					
10	A0A287BRF1	C1q domain-containing protein	LOC110258309	1.71					
11	A0A4X1U1C6	Uncharacterized protein		1.64					
12	A0A287B583	Annexin	ANXA4		2.58				
13	F1RXM6	Thyroxine-binding globulin	SERPINA7		2.52				
14	A0A4X1VDK0	Olfactomedin-like domain-containing protein			Inf				
15	A0A480HB69	Myosin-10		Inf		Inf			
16	A0A1K0H3U3	Globin B1	GLNB1	11.52		5.92			
17	A0A4X1W1F9	Uncharacterized protein				5.43			
18	A0A4X1VQR2	Pentraxin		2.56	2.50	2.90			
19	A0A5G2QEF4	Perilipin 5	PLIN5			2.61			
20	I3LRJ4	Vitamin K-dependent protein C	PROC	1.82		2.36			
21	F2Z5C1	Annexin	ANXA5				3.73		
22	A0A287A0E9	Calcium-activated chloride channel regulator 1	CLCA1				2.14		
23	A0A287A275	Tubulin beta chain	TUBB4B					Inf	
24	A0A4X1U1A1	Ig-like domain-containing protein					Inf	Inf	
25	A0A4X1UVN1	Epimerase domain-containing protein						1.74	
26	A0A287A1B4	Uncharacterized protein	PZP						Inf
27	A0A480VXT1	Alpha-1-antichymotrypsin							Inf
28	A0A4X1VYX8	Fibulin-1							Inf
29	A0A287BIP4	Coagulation factor XII	F12						3.57
30	A0A4X1U6L2	Uncharacterized protein							2.63
31	A0A4X1UAP2	Elongation factor Tu							2.06
32	A0A4X1TZA7	Uncharacterized protein							2.03
33	B3CL06	Transferrin	TF						1.81
34	A0A287B9R5	SERPIN domain-containing protein	SERPINA3-2					1.80	
35	A0A4X1SLE1	Haptoglobin							1.77
36	F1SCC6	SERPIN domain-containing protein	SERPINA3-2					1.70	
37	A0A4X1W9S1	Carbonic anhydrase					2.51		
38	A0A286ZM82	Calumenin	CALU				15.18	3.66	

Inf: The expression level in the denominator group was too low to be tested.

**Table 2:** List of downregulated DEPs in different comparisons.

No.	Protein	Description	Gene	FC					
				ED0/ED-3	ED0/ED3	ED0/ED8	ED-3/ED3	ED-3/ED8	ED3/ED8
1	A0A286ZM82	Calumenin	CALU	0.33					0.24
2	A0A4X1W9S1	Carbonic anhydrase		0.32		0.23			0.28
3	A0A5G2R9V5	Uncharacterized protein							0.31
4	A0A4X1VK11	Uncharacterized protein							0.33
5	A0A5G2R890	Adaptor related protein complex 2 subunit alpha 2	AP2A2						0.34
6	A0A4X1U383	SERPIN domain-containing protein							0.35
7	A0A287BEC5	Transmembrane protease serine	TMPRSS11A			0.62			0.39
8	A0A4X1SHY6	SAM domain-containing protein							0.39
9	K7GRW1	Interleukin 6 signal transducer	IL6ST						0.40
10	A0A4X1UV56	FAM83 domain-containing protein							0.41
11	A0A287AH24	BRO1 domain-containing protein	PDCD6IP						0.41
12	A0A4X1TFG8	NPC intracellular cholesterol transporter 2							0.41
13	A0A287B2P9	Thy-1 cell surface antigen	THY1						0.44
14	O62680	CD59 glycoprotein	CD59						0.48
15	A0A4X1VK10	H15 domain-containing protein							0.49
16	A0A480W0D6	Cystatin-C (Fragment)				0.40			0.49
17	A0A287B8T7	Antileukoproteinase	LOC100512873					0.49	
18	A0A5G2RAS7	IF rod domain-containing protein	LOC100515166					0.50	
19	F1RP76	Sciellin	SCEL					0.26	0.51
20	A0A287AG13	Apolipoprotein B	APOB						0.52
21	A0A5S6GR81	Cathepsin B	CTSB						0.52
22	A0A4X1V609	Amino_oxidase domain-containing protein				0.47			0.52
23	A0A4X1UHH9	Actin-related protein 2/3 complex subunit 5							0.54
24	A0A4X1UZ33	UBIQUITIN_CONJUGAT_2 domain-containing protein							0.55
25	F1S6S9	Proteinase 3	PRTN3					0.47	0.55
26	A0A5S6H0X2	Protein S100-A12	S100A12			0.62			0.56
27	A0A4X1SLI2	Leukotriene A(51) hydrolase							0.56
28	A0A4X1VEI0	Uncharacterized protein				0.37			0.59
29	A0A4X1U5C5	Sulfhydryl oxidase							0.60
30	A0A480IS07	Thrombospondin-1							0.61
31	A0A5G2QXY8	Elongin C	ELOC						0.62
32	A0A287BLX2	Folate receptor alpha	FOLR1						0.62
33	A0A287BBS4	ATP synthase subunit alpha	ATP5F1A						0.63
34	A0A480X6D5	Ribokinase	RBKS					0.30	
35	A0A286ZRS0	Glutathione synthetase	GSS					0.32	
36	Q6YT39	Lactotransferrin	LTF					0.37	
37	A0A481DHP5	40S ribosomal protein S25 (Fragment)						0.43	
38	A0A480M5F4	Cathepsin D preproprotein (Fragment)						0.46	
39	A0A4X1UVN1	Epimerase domain-containing protein		0.50					
40	A0A4X1U1A1	Ig-like domain-containing protein		#					
41	A0A5G2QVV0	Chaperonin containing TCP1 subunit 5	CCT5				0.28		
42	A0A287AQK7	Heat shock protein HSP 90-alpha	HSP90AA1				0.36		
43	A0A4X1V8P2	CN hydrolase domain-containing protein					0.50		
44	A0A480U0E1	Actin, cytoplasmic 1					0.51		
45	A0A5K1TWC1	Apolipoprotein D	APOD				0.63		
46	F1SRC8	C-type lectin domain family 3 member B	CLEC3B				0.65		
47	A0A287AHK1	GC vitamin D binding protein	GC				0.66		
48	F1RN68	Carboxypeptidase D	CPD			0.27			
49	A0A4X1TI96	P-type domain-containing protein				0.27			
50	A0A480KQF0	ATP-dependent (S)-NAD(P)H-hydrate dehydratase	CARKD	0.27		0.29			
51	A0A287B6M0	Carbonic anhydrase	CA2			0.29			
52	A0A4X1V8D9	Uncharacterized protein				0.34			
53	A0A4X1W0G3	Uncharacterized protein				0.36			
54	A0A4X1WD65	IF rod domain-containing protein				0.37			
55	A0A4X1UZC0	Uncharacterized protein				0.42			
56	A0A287B8Z2	Fructose-bisphosphate aldolase	ALDOC		0.28	0.45			
57	A0A4X1TUA6	HMA domain-containing protein				0.45			
58	A0A5G2QVE1	Uncharacterized protein	LOC100739218	0.45		0.47			
59	F2Z4Z1	Tyrosine 3-monooxygenase/tryptophan 5-monooxygenase activation protein gamma	YWHAG			0.49			
60	A0A287A191	Desmocollin 2	DSC2			0.51			
61	A0A286ZQK6	Uncharacterized protein	ZG16B			0.51			
62	F1SSZ0	Arachidonate 15-lipoxygenase type B	ALOX15B			0.51			
63	A0A4X1UXI7	Uncharacterized protein				0.52			
64	K7GNU8	EF-hand domain-containing protein	LOC110260465		0.53				
65	A9GYW6	Carboxylic ester hydrolase (Fragment)	APLE			0.59			
66	A0A4X1UXZ5	Uncharacterized protein				0.62			
67	A0A5G2QI19	Proteasome subunit alpha type	PSMA1			0.62			
68	A0A5G2R6S0	Rac family small GTPase 2	RAC2		0.18				
69	A0A480KNE9	Persulfide dioxygenase ETHE1, mitochondrial isoform 1			0.56				
70	F1SEC5	Uncharacterized protein	CGREF1	0.39					
71	A0A4X1VIL3	Uncharacterized protein		#					
72	A0A287AW90	G protein subunit beta 1	GNB1			#			
73	A0A480MTY5	STE20-like serine/threonine-protein kinase isoform 1				#			
74	A0PFK6	F-actin-capping protein subunit alpha	CP	#		#			
75	A0A287AW99	Intelectin 2	ITLN2				#		
76	A0A287ALP1	Ribonuclease T2	RNASET2				#		
77	A0A287ARC6	UV excision repair protein RAD23	RAD23A				#		
78	A0A5G2QYD9	Cytochrome b5	CYB5A				#		
79	F1RNN0	Vacuolar protein sorting-associated protein 29	VPS29				#		
80	A0A5G2RBM6	Glutaredoxin 3	GLRX3					#	
81	A0A4X1SH17	Uncharacterized protein						#	
82	A5J2A8	Thioredoxin (Fragment)	TRX			#NAME?		#	
83	A0A287AZK2	Transgelin	TAGLN						#

#: The expression level in the numerator group was too low to be tested.

Functional annotation was performed using GO and KEGG analyzes. According to the enrichment analysis (Table 3), the significant GO items generated among different comparisons with ED0 were mainly classified by molecular function, including actin cytoskeleton, calcium ion binding, ADP-dependent NAD(P)H-hydrate dehydratase activity, glycolytic process, fructose-bisphosphate aldolase activity, calcium-dependent phospholipid binding, and lyase activity. In the KEGG enrichment analysis (Table 4), the significant pathways were mainly related to the peroxisome proliferator-activated receptors (PPAR) signaling pathway, epidermal growth factor receptor (EGFR) tyrosine kinase inhibitor resistance, prolactin signaling pathway, vascular endothelial growth factor (VEGF) signaling pathway, Wnt signaling pathway, cAMP signaling pathway, nitrogen metabolism, and serotonergic synapse.

**Table 3:** GO items significantly differently enriched among different comparisons.

Comparison	GO_Term	GO_Class	*P* value	Protein ID
ED0/ED-3	Actin cytoskeleton	CC	0.026015581	A0A480HB69 A0PFK6
	Cytoskeletal part	CC	0.046860563	A0A480HB69 F1RKM0 A0PFK6
	Calcium ion binding	MF	0.023151248	I3LRJ4 A0A4X1UBJ8 1SEC5 A0A286ZM82
	ADP-dependent NAD(P)H-hydrate dehydratase activity	MF	0.02640264	A0A480KQF0
ED0/ED3	Glycolytic process	BP	0.029458033	A0A287B8Z2
	Fructose-bisphosphate aldolase activity	MF	0.009884625	A0A287B8Z2
	Calcium-dependent phospholipid binding	MF	0.019703897	A0A287B583
ED0/ED8	Metal ion transport	BP	0.03630363	A0A4X1TUA6
	Protein phosphorylation	BP	0.03630363	A0A480MTY5
	Actin cytoskeleton	CC	0.047481237	A0A480HB69 A0PFK6
	Lyase activity	MF	0.003696926	A0A287B8Z2 A0A480KQF0
	ADP-dependent NAD(P)H-hydrate dehydratase activity	MF	0.03630363	A0A480KQF0
	Protein serine/threonine kinase activity	MF	0.03630363	A0A480MTY5
ED-3/ED3	Response to stress	BP	0.026533829	A0A287AQK7 A0A287ARC6
	Nucleotide-excision repair	BP	0.029506587	A0A287ARC6
	Proteasome-mediated ubiquitin-dependent protein catabolic process	BP	0.029506587	A0A287ARC6
	Ribonuclease T2 activity	MF	0.014851485	A0A287ALP1
	Organic cyclic compound binding	MF	0.024004408	A0A287AQK7 A0A5G2QVV0 A0A287ALP1 A0A287ARC6
	Heterocyclic compound binding	MF	0.024004408	A0A287AQK7 A0A5G2QVV0 A0A287ALP1 A0A287ARC6
	Damaged DNA binding	MF	0.029506587	A0A287ARC6
ED-3/ED8	Cell redox homeostasis	BP	0.00791243	A5J2A8 A0A5G2RBM6
	Glutathione biosynthetic process	BP	0.026249898	A0A286ZRS0
	Glutathione synthase activity	MF	0.01320132	A0A286ZRS0
	Catalytic activity	MF	0.014907079	A0A287A275 A0A4X1UVN1 F1S6S9 A0A480M5F4 A0A286ZRS0 A0A5G2RBM6
	Aspartic-type endopeptidase activity	MF	0.026249898	A0A480M5F4
	Protein disulfide oxidoreductase activity	MF	0.026249898	A0A5G2RBM6
ED3/ED8	Nucleotide catabolic process	BP	0.04620462	A0A4X1VK11
	Nucleosome assembly	BP	0.04620462	A0A4X1VK10
	Extracellular region	CC	0.038882853	A0A4X1TZA7 A0A4X1U6L2 A0A287A1B4 A0A287BIP4 A0A480IS07 A0A287B8T7
	Nucleosome	CC	0.04620462	A0A4X1VK10
	Peptidase inhibitor activity	MF	0.033979832	A0A4X1TZA7 A0A4X1U6L2 A0A287A1B4 A0A480W0D6 A0A287B8T7
	Lipid transporter activity	MF	0.04620462	A0A287AG13
	Thiol oxidase activity	MF	0.04620462	A0A4X1U5C5

**Table 4:** List of pathways significantly differently enriched among different comparisons.

Comparison	KEGG pathway	*P* value	Protein ID
ED0/ED-3	PPAR signaling pathway	0.0139	A0A076KWW8 A0A287BRF1
	EGFR tyrosine kinase inhibitor resistance	0.0193	B6E241
	Prolactin signaling pathway	0.0193	B6E241
	Breast cancer	0.0193	B6E241
	Endocrine resistance	0.0383	B6E241
	ErbB signaling pathway	0.0383	B6E241
	Osteoclast differentiation	0.0383	B6E241
	Adipocytokine signaling pathway	0.0383	A0A287BRF1
	Hepatitis C	0.0383	B6E241
	Endometrial cancer	0.0383	B6E241
	Chronic myeloid leukemia	0.0383	B6E241
	Non-small cell lung cancer	0.0383	B6E241
	Choline metabolism in cancer	0.0383	B6E241
ED0/ED3	VEGF signaling pathway	0.0116	A0A5G2R6S0
	Pancreatic cancer	0.0116	A0A5G2R6S0
	Choline metabolism in cancer	0.0116	A0A5G2R6S0
	Colorectal cancer	0.0173	A0A5G2R6S0
	Wnt signaling pathway	0.0230	A0A5G2R6S0
	cAMP signaling pathway	0.0287	A0A5G2R6S0
	Sphingolipid signaling pathway	0.0344	A0A5G2R6S0
	Axon guidance	0.0344	A0A5G2R6S0
	Fructose and mannose metabolism	0.0457	A0A287B8Z2
	Thyroid hormone synthesis	0.0457	F1RXM6
ED0/ED8	Nitrogen metabolism	0.0081	A0A4X1W9S1 A0A287B6M0
	Serotonergic synapse	0.0081	F1SSZ0 A0A287AW90
ED-3/ED3	Pantothenate and CoA biosynthesis	0.0116	A0A4X1V8P2
	Protein processing in endoplasmic reticulum	0.0254	A0A287AQK7 A0A287ARC6
	Nucleotide excision repair	0.0344	A0A287ARC6
	Th17 cell differentiation	0.0344	A0A287AQK7
	Progesterone-mediated oocyte maturation	0.0344	A0A287AQK7
	Nitrogen metabolism	0.0457	A0A4X1W9S1
ED3/ED8	Endocytosis	0.0127	A0A287BLX2 A0A287AH24 A0A4X1UHH9 A0A5G2R890
	Cholesterol metabolism	0.0318	A0A287AG13 A0A4X1TFG8
	HIF-1 signaling pathway	0.0413	B3CL06 A0A5G2QXY8

### Relative expression verification by PRM

**Figure 4:**
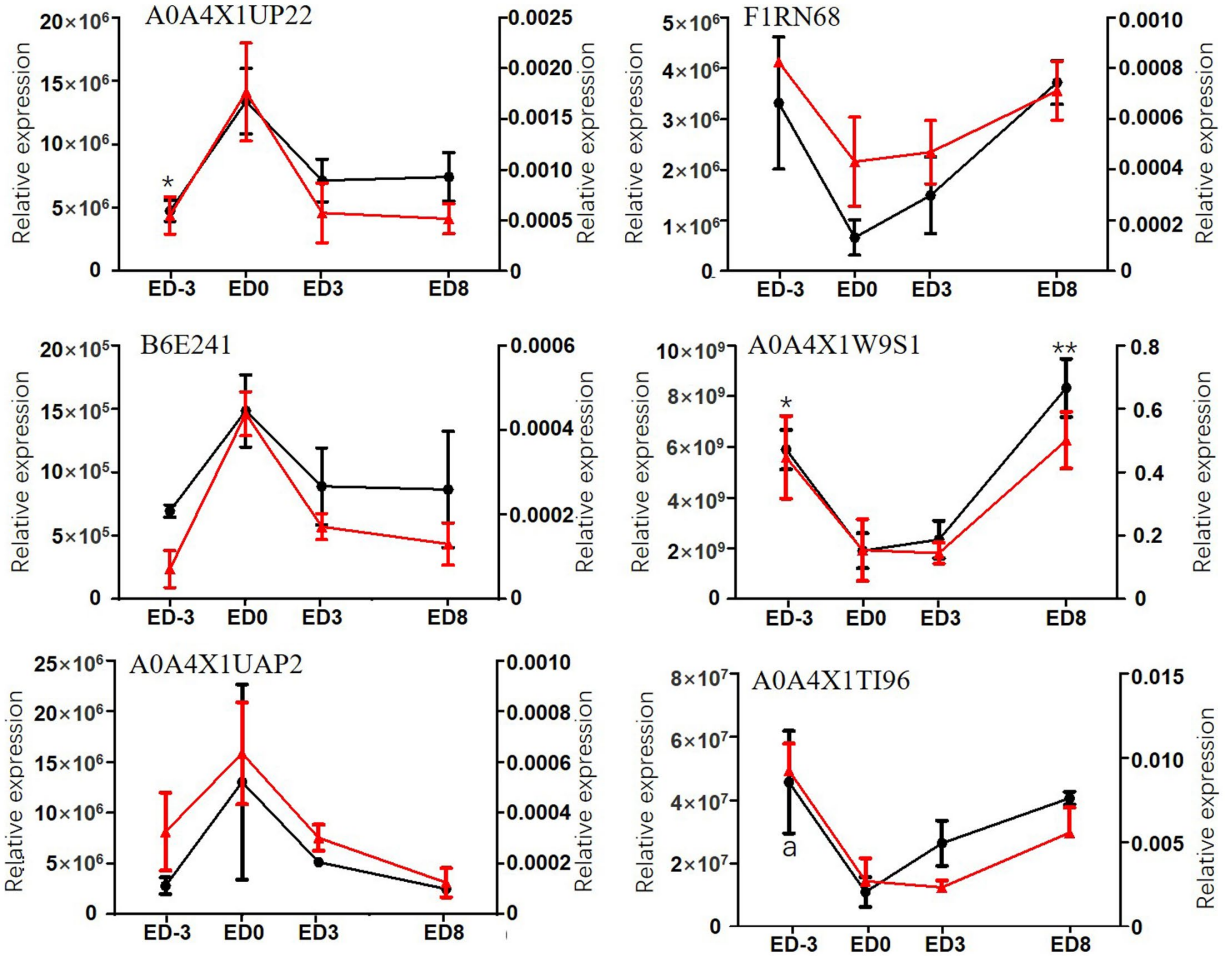
Relative expression levels of six DEPs assayed by label-free method and PRM in pig saliva during the estrous cycle. The primary coordinate represents label-free assay, left, red lines; * and ** indicate significant differences compared with ED0, *p* < 0.05, *p* < 0.01, respectively. The secondary coordinate represents PRM assay, right, black lines; a represents the significant difference compared with ED0.

The 12-sample (3 replicates) cohort derived from four representative time-points of the estrous cycle was sent for PRM assay to verify the relative expression. PRM quantitative analysis of DEPs exhibited high agreement with the label-free results (Figure 4). The relative expression trends of six proteins, including stomatin-domain-containing protein (STOM, A0A4X1UP22), growth factor receptor bound protein 2 (Grb2, B6E241), elongation factor Tu (EFTU, A0A4X1UAP2), carboxypeptidase D (CPD, F1RN68), carbonic anhydrase (CA, A0A4X1W9S1), and trefoil factor 3 (TFF3, A0A4X1TI96), were validated using the PRM method and exhibited similar expression trends as label-free. The first three proteins exhibited an upgrade, followed by a descending trend, with a maximum expression level of 1.34 × 10^7^, 1.48 × 10^6^, 1.30 × 10^7^, respectively, for label-free analysis, and 0.0017, 0.00044, 0.00063, respectively, for PRM analysis, at estrus. Instead, the other three proteins showed a significant ‘V’ trend, with the lowest level of 6.6 × 10^5^, 1.91 × 10^9^, 1.10 × 10^7^, respectively, for label-free analysis, and 0.00043, 0.15, 0.0027, respectively, for PRM analysis, appearing at estrus.

## Discussion

The present study focused on characterizing the overall proteomics during the estrous cycle in pig saliva and searched for estrus relevant or specific proteins. Given the evident advantages of saliva in protein species diversity, high abundance, and immense popularity in biological diagnosis, it is reasonable to hypothesize that exploring protein profiles and estrus-specific proteins in saliva may assist in developing novel non-invasive estrus diagnostic methods. Recent data showed that 1,155 total proteins were found in four representative groups, and up to 115 proteins were differentially expressed among the different groups. Both the total number and DEPs were larger than those obtained by Li (2), which may be caused by different breeds and comparisons. PRM quantity validated the expression patterns of the six proteins including stomatin-domain-containing protein, growth factor receptor bound protein, elongation factor Tu, carboxypeptidase, carbonic anhydras, and trefoil factor 3, displaying consistent expression trends with the label-free method.

Protein levels changed over time during the estrous cycle. In this study, many DEPs were upregulated at estrus, such as C-type lectin domain family 8 member A, STOM, Grb2, annexin A4, thyroxine-binding globulin, globin B1, pentraxin, perilipin 5 (Plin5), Vitamin K-dependent protein C. The expression of pentraxin in cumulus cells is positively correlated with *in vivo* fertilization by affecting the expansion of the cumulus oophorus, the quality of the corresponding oocytes, and ovulation (25). Plin5 bidirectionally regulates the dynamic balance of lipid metabolism in oxidative tissues by controlling the activity of adipose triglyceride lipase. Plin5 inhibits the lipolysis in the basic state, while the suppressed lipolysis process would be accelerated once the stress occurred (26). However, there are few studies regarding the function of the other upregulated proteins at estrus.

Notably, the expressions of many proteins were downregulated during diestrus. Carbonic anhydrase (CA), as a zinc-containing enzyme, is widely distributed in mammal tissues, including the reproductive tract (27). CA plays key roles in body fluids balance by influencing metabolic carbon dioxide transport (28).LTF is a major component of mucosal fluids defense because of the antimicrobial and immunomodulating properties. It is evident that the bimodal distribution of LTF in cervical immune components is determined by the menstrual cycle (29). Besides, LTF is expressed correlated with the circulating level of 17β-estradiol (E_2_), and selectively synthesized in the uterine epithelium dependent on estrogen receptor α (ERα) (30). For several other downregulated proteins during diestrus, e.g., calumenin, SERPIN domain-containing protein, sciellin, ribokinase, glutathione synthetase, few reports are available for their roles in reproduction.

GO function classification of DEPs established their close association with biological process, cellular components, and molecular function. Calcium ion binding was enriched in the greatest number of proteins in the ED0 vs. ED-3 group. As a second intracellular messenger, calcium ions are crucial in cellular physiological processes, and are essential for mammalian oocyte growth, meiosis, hormone production, growth factor secretion, and meiotic maturation (31). In this category, the four enriched proteins, I3LRJ4, A0A4X1UBJ8, 1SEC5, and A0A286ZM82, suggested their involvement in oocyte maturation. The actin cytoskeleton contributes to oocyte meiotic maturation by mediating spindle assembly, length and chromosome segregation (32). Overlapping of the actin cytoskeleton in the ED0 vs. ED8 and ED0 vs. ED-3 groups emphasized their importance during the estrus stage.

KEGG enrichment analysis of DEPs was performed to identify the functional pathways during the reproductive cycle. Compared with ED0, the most enriched pathways included the PPAR signaling pathway, EGFR tyrosine kinase inhibitor resistance, prolactin signaling pathway, VEGF signaling pathway, Wnt signaling pathway, cAMP signaling pathway, nitrogen metabolism, and serotonergic synapse. The PPAR pathway was demonstrated to be pivotal in canine gametogenesis, ovulation, or CL regression during the estrous cycle (33). VEGF signaling is a key pathway for granulosa cell proliferation, steroid hormone synthesis, apoptosis inhibition, and ovarian angiogenesis during follicular development and ovulation (34). The Wnt pathway is required for embryonic and adult ovarian development. Wnt-4 stimulates granulosa cell differentiation and female sexual development during the embryonic stage. In contrast, adult ovarian function and fertility rely on the synchronized actions of Wnt family genes and hormones (35). The inhibitory effects of nitric oxide (NO), a representative product of nitrogen metabolism, on steroidogenesis have been reported in several animal models. The controversial effects of NO during folliculogenesis, which protects or induces follicular apoptosis, were concentration-dependent. Moreover, the NO pathway exerts a central influence on mammalian oocyte meiotic maturation among the numerous mediator molecules (36). The strong relationship between these pathways and reproductive physiology strengthens the reliability of our enrichment results.

Both the label-free and PRM groups consistently revealed the maximum levels of STOM, Grb2 and EFTU during estrus. There are five members in the stomatin-domain-containing protein family: STOM, STOML1, STOML2, STOML3, and podocin (37). The proliferation and invasion of human trophoblasts can be mediated by STOML2 through modulating mitochondrial function (38). A significant increase in STOML2 expression could promote endometrial stromal cell proliferation and decidualization in mice and human (39). Grb2 induces various cellular events as an adaptor protein in signal transduction pathways (40). The Grb2/Sos complex activates Ras in response to cellular growth factors. Grb2 is involved in vulvar development and sex myoblast migration in *Caenorhabditis. elegans* (40). In *Xenopus* oocytes, Grb2 induced the re-initiation of meiosis (40). A study identified the Grb2 as a key candidate gene in progesterone production during ovulation through bioinformatics analysis and verified its function in progestogenic pathways in rat COCs (41). In the current study, Grb2 showed maximum expression during estrus, which might be related to ovulation. EFTU increased from day 12 to 16 in corpus luteum of cyclic ewes (42), which may assist corpus luteum regression during estrus. In contrast, EFTU was most abundant on the day of estrus in our study, indicating its potential effects on follicular development, ovulation, and LH synthesis. This distinct tendency may be attributed to different organisms or tissues.

Simultaneously, minimal expression of CPD, CA and TFF3 was observed in label-free and PRM. CPD is a carboxypeptidases (CPs) family member characterized by a conserved metallocarboxypeptidase domain. The CPs family in mammals includes CPH, CPM, CPE, and nine other proteins (43). CPD signals are significantly high in bovine dominant follicles and are considered a reliable marker for dominant follicles (44). CPD has great functional and structural similarities with other CPs (45); however, limited studies are available on CPD function in reproduction (44, 46). In our study, CPD showed the lowest level of saliva on estrus day, suggesting that CPD may regulate estrus physiology by modulating the precursors processes of hormones and neuropeptides. A previous study explored the localization of CA in the genitalia of female pigs. CA was positive in the blood vessel endothelium and absent in the ovarian parenchyma. In the oviduct, conspicuous CA activity was observed in the surface epithelium of the tubal ampulla, where fertilization and early embryo development occurred. Moreover, CA was stained in the uterotubal junction-tubal isthmus, which acts as a sperm reservoir. Both the intensity and localization of CA remained constant during the estrous cycle. The localization of CA may indicate its crucial role in maintaining acid–base homeostasis in the luminal fluid (47). Likewise, the involvement of CA in bicarbonate secretion during the estrous cycle was also observed in mouse and rat uterine (48, 49) since carbonic anhydrase 2(CAR2, CA2) had the maximum expression at estrus during the estrous cycle when the resting uterine surface pH was significantly higher than that at diestrus; nevertheless, the high pH could be reduced significantly by the CA inhibitor (48). Remarkably, estrogen induced a parallel increase in CAR2 expression and endometrial surface pH. Our data showed the opposite result; CA had the lowest expression at estrus. Hence, the expression pattern of CA may change with species. In human, the expression of TFF3 is upregulated on day 4 vs. Day 2 of menstruation and is implicated in endometrial regeneration and repair during menstruation (50). The presence of TFF3 in saliva may be associated with the endometrium, whereas the underlying function and mechanisms of estrus are not yet known and need to be resolved.

## Conclusion

The present study compared global saliva proteome profiles during the estrous cycle based on the label-free quantitative proteomics in a crossbred pig. The identification and enrichment analysis of the differentially expressed proteins indicated the involvement of many proteins in pig estrus physiological functions. The expression trends of the six proteins were confirmed using PRM. This study provided new insights into the physiology of the reproductive cycle and served as a reference for developing estrus detection kits or strips. Nevertheless, further investigations are needed to uncover the confusion regarding DEPs generation, secretion, function mechanisms, and practical potential in estrus detection.

## Data availability statement

The data presented in the study are deposited in the ProteomeXchange Consortium repository, accession number PXD043141.

## Author contributions

HX conceived the study, designed and performed the experiments, conducted data analysis, prepared figures and tables, and wrote the manuscript. BL, BH, and YW prepared materials and collected samples. SW and FM modified the manuscript. JL supervised the study. All authors contributed to the article and approved the submitted version.

## Footnotes

^1^https://jvenn.toulouse.inrae.fr/app/example.html

^2^http://string.embl.de/
